# An observational study to investigate the relationship between plasma glucosylsphingosine (lyso-Gb1) concentration and treatment outcomes of patients with Gaucher disease in Japan

**DOI:** 10.1186/s13023-022-02549-6

**Published:** 2022-11-03

**Authors:** Hiroyuki Ida, Yuko Watanabe, Rieko Sagara, Yoichi Inoue, Jovelle Fernandez

**Affiliations:** 1 The Jikei University Hospital, Tokyo, Japan; 2 Japan Medical Office, Takeda Pharmaceutical Company Limited, 1-1, Nihonbashi-Honcho 2-Chome, Chuo-Ku, Tokyo, 103-8668 Japan

**Keywords:** Biomarkers, Gaucher disease, Lyso-Gb1 (glucosylsphingosine), Lyso-GL1 (glucosylsphinogosine), Japan, Velaglucerase alfa

## Abstract

**Background:**

Gaucher disease (GD) is an autosomal recessive disease caused by *GBA1* mutations resulting in glucosylceramide accumulation in macrophages. GD is characterized by hepatosplenomegaly, anemia, thrombocytopenia, bone complications, and neurological complications. Glucosylsphingosine (lyso-Gb1), a deacylated form of glucosylceramide, has been identified as a promising biomarker for the diagnosis and treatment response in GD. The aim of this study was to examine the relationship between plasma lyso-Gb1 and therapeutic goals for GD (improvements in hepatomegaly, splenomegaly, anemia, thrombocytopenia, bone pain, and bone crisis), as well as disease type and *GBA1* mutation type, in Japanese patients with GD receiving velaglucerase alfa, an enzyme replacement therapy (ERT). Furthermore, this study compared the plasma lyso-Gb1 concentration observed in Japanese patients included in this study with that observed in a previous non-Japanese clinical study.

**Results:**

This non-interventional, open-label, multicenter observational cohort study (October 2020 to March 2021) included a total of 20 patients (of any age) with GD (type 1: n = 8; type 2: n = 9; type 3: n = 3) treated with velaglucerase alfa for ≥ 3 months. Median (minimum–maximum) duration of velaglucerase alfa treatment was 49.5 (3–107) months. A total of 14 (70.0%) patients achieved all therapeutic goals (i.e., 100% achievement; improvements in hepatomegaly, splenomegaly, anemia, thrombocytopenia, bone pain, and bone crisis). Overall, median (minimum–maximum) lyso-Gb1 concentration was 24.3 (2.1–150) ng/mL. Although not statistically significant, numerically lower plasma lyso-Gb1 concentrations were observed in patients with 100% achievement compared with those without; no statistically significant difference in plasma lyso-Gb1 concentration was observed between patients with different disease type or mutation type. Furthermore, lyso-Gb1 concentrations observed in Japanese patients were numerically lower than that observed in a previous study of non-Japanese patients with GD receiving ERT.

**Conclusions:**

In this study, high achievement rates of therapeutic goals with low lyso-Gb1 concentration were observed, demonstrating a correlation between therapeutic goals and lower plasma lyso-Gb1 concentration in Japanese patients with GD treated with velaglucerase alfa. This study further suggests that plasma lyso-Gb1 concentration may be a useful biomarker for treatment response in patients with GD.

**Supplementary Information:**

The online version contains supplementary material available at 10.1186/s13023-022-02549-6.

## Background

Gaucher disease (GD) is an autosomal recessive genetic disease that is caused by *GBA1* gene mutations. Such mutations result in the deficiency in the β-glucocerebrosidase (GBA) enzyme, and the accumulation of its substrate glucosylceramide, in macrophages forming pathologic Gaucher cells [1, 2]. Patients with GD present with a range of progressive and debilitating signs and symptoms, including hepatosplenomegaly, anemia, thrombocytopenia, bone complications, and neurological complications [3]. GD is classified into 3 major clinical subtypes based on the presence and the extent of neurological involvement; type 1 is non-neuronopathic GD, type 2 is acute neuronopathic GD, and type 3 is subacute/chronic neuronopathic GD [4]. In Japan, the prevalence of GD is estimated to be 1 in 330,000 patients [5]; approximately 211 patients in Japan are currently estimated to have GD [6]. Current treatments in Japan include enzyme replacement therapy (ERT) and substrate reduction therapy; ERT is recommended as a standard of care for all types of GD, regardless of age and disease types [5]. Among the Japanese patients with GD, the L444P mutation, which is associated with a more severe disease, is most common [7–9], unlike the Western European, American, and Jewish populations, where the N370S mutation is most common [10].

Several markers including angiotensin-converting enzyme [11], chitotriosidase and chemokine ligand 18 (CCL18) have been used for diagnosis and disease monitoring to evaluate treatment efficacy for GD [12]. Both chitotriosidase and CCL18 are secreted by activated macrophages, including Gaucher cells, and are found in markedly increased levels in patients with GD [13, 14]. Their levels have been shown to decrease with treatments such as ERT [13–16]; however, neither of these markers has a direct role in the pathophysiology of the disease and neither is specific to GD. In fact, approximately 6% of individuals have deficient chitotriosidase activity due to a homozygous mutation in the chitotriosidase gene [17] and, although to a lesser extent, both markers are also increased in other diseases and conditions [2]. Therefore, a biomarker that is sensitive and specific to GD would be more beneficial for diagnosis and evaluation of treatment response, and to aid in deciding treatment strategies in GD.

Glucosylsphingosine (lyso-Gb1), a deacylated form of glucosylceramide, has been shown to have a pathophysiologic role in GD [18]. Elevated lyso-Gb1 levels are observed in patients with GD [19, 20]; however, ERT is known to markedly reduce lyso-Gb1 levels [21–24]. Several studies have also demonstrated that the lyso-Gb1 level correlates with chitotriosidase, CCL18, and various GD clinical symptoms [21–24]. Therefore, evidence suggests that lyso-Gb1 is a promising, reliable biomarker with greater sensitivity and specificity for GD compared with chitotriosidase and CCL18 [19]. However, little is known about the relationship between lyso-Gb1 levels and the achievement rate of various therapeutic goals of GD. Furthermore, no information on lyso-Gb1 concentrations in Japanese patients with GD is currently available.

This study aimed to examine the relationship between plasma lyso-Gb1 concentration and treatment outcome, evaluated by the overall achievement rate of therapeutic goals (improvements in hepatomegaly, splenomegaly, anemia, thrombocytopenia, bone pain, and bone crisis) in Japanese patients with GD who were treated with velaglucerase alfa. Further, the relationships between plasma lyso-Gb1 concentration and disease type and mutation type were also assessed.

## Results

### Demographic and baseline clinical characteristics

A total of 20 patients with GD who were treated with velaglucerase alfa for ≥ 3 months were included in the study (Table 1). Median (min–max) age was 14.0 (2–74) years, and 55% of patients were male. There were 8 (40%) patients with type 1, 9 (45%) patients with type 2, and 3 (15%) patients with type 3 GD. Median (min–max) duration of disease was 9.0 (1–50) years, with median (min–max) duration of velaglucerase alfa treatment of 49.5 (3–107) months. Furthermore, the most common *GBA* gene mutation was the L444P mutation (13/20 patients; 65%); 3 (15.0%) were L444P/L444P genotype, and 10 (50%) were L444P/others genotype. Neurological symptoms were present in 13 (65.0%) patients, and bone symptoms were present in 5 (25.0%) patients.

**Table 1:** Patient demographic and baseline characteristics

Characteristics	N = 20
Age, years^a^	
Median (min–max)	14.0 (2–74)
Sex, n (%)	
Male	11 (55.0)
Female	9 (45.0)
Onset of disease, years^b^	
Median (min–max)	2.0 (1–70)
Duration of disease, years^b^	
Median (min–max)	9.0 (1–50)
Disease type, n (%)	
Type 1	8 (40.0)
Type 2	9 (45.0)
Type 3	3 (15.0)
Treatment history, n (%)^c^	
Enzyme replacement therapy	20 (100.0)
Bone marrow transplantation	0 (0)
Substrate reduction therapy	1 (5.0)
Chemical chaperone therapy	5 (25.0)
Others	1 (5.0)
Duration of velaglucerase alfa treatment, months	
Mean (SD)	49.6 (28.7)
Median (min–max)	49.5 (3–107)
Concomitant ambroxol use, n (%)	5 (25.0)
*GBA* gene mutation, n (%)	
L444P mutation	13 (65.0)
F213I mutation	2 (10.0)
D4409H mutation	2 (10.0)
R463C mutation	0 (0)
Other	3 (15.0)
*GBA* genotype, n (%)	
L444P/L444P	3 (15.0)
L444P/others	10 (50.0)
Other genotype	7 (35.0)
Presence of comorbidity, n (%)	12 (60.0)
Complications, n (%)	
Neurologic symptoms	13 (65.0)
Bone symptoms	5 (25.0)
Other	15 (75.0)

^a^Age at the time of enrollment

^b^Onset of disease was calculated as the time between date of birth (year) and date of diagnosis (year); duration of disease was calculated as the time between date of diagnosis (year) and date of informed consent acquisition (year)

^c^Patients could have received multiple treatments

*GBA*, β-glucocerebrosidase gene; max, maximum; min, minimum; SD, standard deviation

### Achievement of therapeutic goals

A total of 14 (70.0%) patients achieved all 6 therapeutic goals (i.e., had 100% achievement) (Table 2). Seventeen (85.0%) and 18 (90.0%) patients reached the therapeutic goals (score of 0) for hepatomegaly and splenomegaly assessments, respectively (Table 2; Additional file 1: Fig. S1). Most patients achieved the therapeutic goals for anemia (17/20; 85.0%) and thrombocytopenia (18/20; 90.0%), with median (min–max) hemoglobin level of 12.5 (7.8–15.9) g/dL and median (min–max) platelet count of 233 (24–473) × 10^3^/μL. Bone pain was absent or very mild, or mild, in 18 (90.0%) patients, and no patients had a bone crisis.

**Table 2:** Summary of achievement of therapeutic goals

Therapeutic goal	N = 20
Number of therapeutic goals achieved	
≤ 1	0 (0)
2	1 (5.0)
3	0 (0)
4	3 (15.0)
5	2 (10.0)
6	14 (70.0)
Hepatomegaly score, achieved (score = 0)	17 (85.0)
0	17 (85.0)
1	3 (15.0)
2	0 (0)
3	0 (0)
Splenomegaly score, achieved (score = 0)	18 (90.0)
0	18 (90.0)
1	1 (15.0)
2	0 (0)
3	1 (5.0)
Anemia, hemoglobin count (g/dL), n	20
Achieved^a^	17 (85.0)
Mean (SD)	12.7 (1.9)
Median (min–max)	12.5 (7.8–15.9)
Thrombocytopenia, platelet count (× 10^3^/μL), n	20
Achieved^b^	18 (90.0)
Mean (SD)	248.2 (102.4)
Median (min–max)	233.0 (24.0–473.0)
Bone pain, achieved (absent or very mild, or mild)	18 (90.0)
Absent or very mild pain	17 (85.0)
Mild pain	1 (5.0)
Moderate pain	2 (10.0)
Severe pain	0 (0)
Intolerable pain	0 (0)
Bone crisis, achieved (absent)	20 (100)
Absent	20 (100)
Present	0 (0)

Data are n (%), unless otherwise stated

^a^Treatment goal for anemia was achieved if hemoglobin level was ≥ 11.0 g/dL for children ≤ 12 years and female ≥ 13 years, and ≥ 12.0 g/dL for males ≥ 13 years

^b^Treatment goal for thrombocytopenia was achieved if platelet count was > 120 × 10^3^/μL for those with platelet count ≥ 60 × 10^3^/μL at the first infusion of velaglucerase alfa or if no data at the first velaglucerase alfa infusion were available; or ≥ 2 times the platelet count at first velaglucerase alfa infusion for those with platelet count < 60 × 10^3^/μL at the first infusion of velaglucerase alfa

max, maximum; min, minimum; SD, standard deviation

### Correlation of plasma lyso-Gb1 concentration and therapeutic goals

**Fig. 1:**
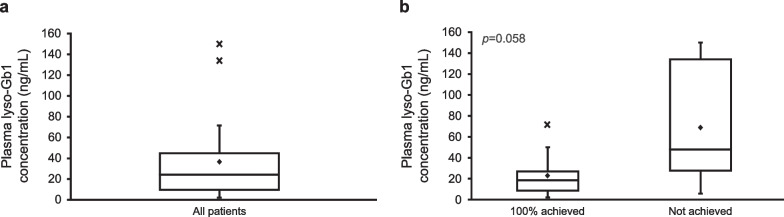
Plasma lyso-Gb1 concentration in **a** all patients, and **b** by achievement of therapeutic goals (100% achieved: achieved all 6 therapeutic goals; Not achieved: did not achieve all 6 therapeutic goals). The bottom of the box represents the first quartile, the top of the box represents the third quartile, and the line in the middle represent the median. The whiskers represent the highest and the lowest value that are not outliers. The diamond symbol represents the mean, and the cross symbol represents outliers. lyso-Gb1, glucosylsphingosine

**Fig. 2:**
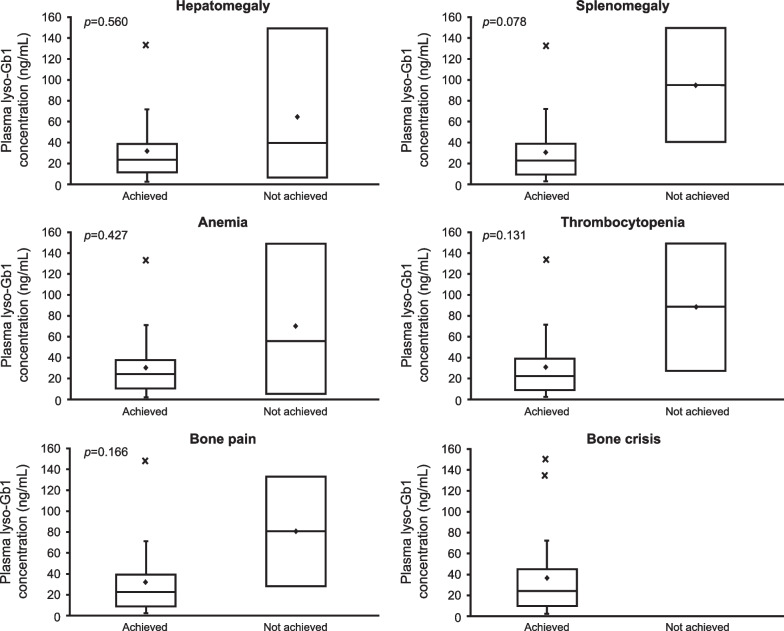
Plasma lyso-Gb1 concentration by achievement of each therapeutic goal. The bottom of the box represents the first quartile, the top of the box represents the third quartile, and the line in the middle represent the median. The whiskers represent the highest and the lowest value that are not outliers. The diamond symbol represents the mean, and the cross symbol represents outliers. lyso-Gb1, glucosylsphingosine

Overall, the median (min–max) plasma lyso-Gb1 concentration was 24.3 (2.1–150) ng/mL (Fig. 1a), with 2 patients having a plasma lyso-Gb1 concentration ≥ 100 ng/mL. Of these 2 patients, one had a lyso-Gb1 concentration of 134 ng/mL and an achievement rate of 83.3% (i.e., achieved 5 therapeutic goals). Another patient had a lyso-Gb1 concentration of 150 ng/mL, with an achievement rate of 33.3% (i.e., achieved 2 therapeutic goals). In patients who achieved all therapeutic goals (had a 100% achievement rate), the median (min–max) plasma lyso-Gb1 concentration was 18.4 (2.1–71.6) ng/mL, and in those who did not achieve all therapeutic goals (did not have 100% achievement rate), the median (min–max) was 48.0 (5.7–150) ng/mL. Although not statistically significant (*p* = 0.058), plasma lyso-Gb1 concentration was numerically lower in patients with 100% achievement compared with those without (Fig. 1b). Furthermore, for each therapeutic goal, numerically lower median plasma lyso-Gb1 concentrations were also observed in patients who achieved the therapeutic goal than in those who did not, except that all patients had no bone crisis (Fig. 2).

### Plasma lyso-Gb1 concentration by GD type

**Fig. 3:**
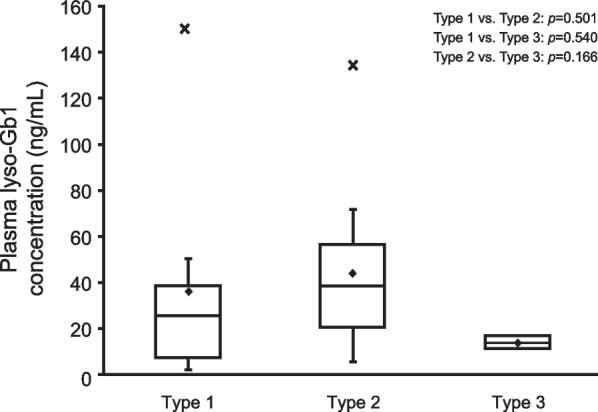
Plasma lyso-Gb1 concentration by disease type. The bottom of the box represents the first quartile, the top of the box represents the third quartile, and the line in the middle represent the median. The whiskers represent the highest and the lowest value that are not outliers. The diamond symbol represents the mean, and the cross symbol represents outliers. lyso-Gb1, glucosylsphingosine

The median (min–max) plasma lyso-Gb1 concentration was 25.6 (2.1–150) ng/mL in 8 patients who had type 1 GD, 38.6 (5.7–134) ng/mL in 9 patients who had type 2 GD, and 13.8 (10.9–16.7) ng/mL in 3 patients who had type 3 GD (Fig. 3). There was no statistically significant difference in plasma lyso-Gb1 concentration between the 3 disease types (type 1 vs. type 2: *p* = 0.501; type 1 vs. type 3: *p* = 0.540; type 2 vs. type 3: *p* = 0.166).

### Plasma lyso-Gb1 concentration by *GBA* gene mutation

**Fig. 4:**
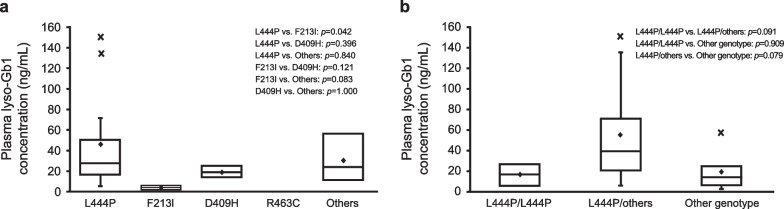
Plasma lyso-Gb1 concentration by type of **a** *GBA1* gene mutation, and **b** genotype. The bottom of the box represents the first quartile, the top of the box represents the third quartile, and the line in the middle represent the median. The whiskers represent the highest and the lowest value that are not outliers. The diamond symbol represents the mean, and the cross symbol represents outliers. Note: No patients were positive for R463C mutation. lyso-Gb1, glucosylsphingosine

In patients who had a L444P mutation (n = 13), F213I mutation (n = 2), or D409H mutation (n = 2), median (min–max) plasma lyso-Gb1 concentration was 27.7 (5.7–150) ng/mL, 3.9 (2.1–5.7) ng/mL, and 19.1 (13.8–24.4) ng/mL, respectively (Fig. 4a). The median (min–max) plasma lyso-Gb1 concentration was 16.7 (5.7–27), 39.0 (6.3–150), and 13.8 (2.1–56.7) ng/mL in patients with L444P/L444P, L444P/others, and other genotypes, respectively (Fig. 4b). There was also no statistically significant difference in plasma lyso-Gb1 concentration between patients with L444P/L444P and L444P/other genotypes (*p* = 0.091).

### Plasma lyso-Gb1 concentration in comparison with previous data of non-Japanese patients with GD

Median (min–max) plasma lyso-Gb1 concentrations observed in this study for the total study population (24.30 [2.12–150] ng/mL) and in patients with type 1 GD (25.60 [2.12–150] ng/mL) were numerically lower compared with treatment-naive (treatment-naive for ≥ 12 months prior to velaglucerase alfa treatment; 43.6 ng/mL at week 209 of velaglucerase alfa treatment) and switch-treatment (treated with imiglucerase for ≥ 2 years prior to velaglucerase alfa treatment; 26.6 ng/mL at week 161 of velaglucerase alfa treatment) non-Japanese patients with type 1 GD (Table 3) [21].

**Table 3:** Comparison of plasma lyso-Gb1 concentrations with global report

Plasma lyso-Gb1 concentration (ng/mL)	This study^a^				Elstein et al. [21]	
	GD type			Total	Treatment-naive^b^ type 1	Switch-treatment^c^ type 1
	Type 1	Type 2	Type 3			
	(n = 8)	(n = 9)	(n = 3)	(N = 20)	(N = 22)	(N = 21)
Median	25.60	38.60	13.80	24.30	43.6^d^	26.6^e^
Min	2.12	5.72	10.9	2.12	–	–
Max	150	134	16.7	150	–	–

^a^Median (min–max) duration of velaglucerase alfa treatment was 49.5 (3–107) months

^b^Treatment-naive patients were defined as those who did not receive ERT treatment for ≥ 12 months prior to velaglucerase alfa treatment

^c^Switch-treatment patients were defined as those who received imiglucerase for ≥ 2 years prior to velaglucerase alfa treatment

^d^Lyso-Gb1 concentration at week 209 of velaglucerase alfa treatment

^e^Lyso-Gb1 concentration at week 161 of velaglucerase alfa following imiglucerase

ERT, enzyme replacement therapy; GD, Gaucher disease; lyso-Gb1, glucosylsphingosine; max, maximum; min, minimum

## Discussion

This is the first observational cohort study to report the plasma lyso-Gb1 concentrations in Japanese patients with GD treated with velaglucerase alfa, and to investigate the relationship of lyso-Gb1 concentration with treatment outcomes, evaluated by achievement rate of multiple GD therapeutic goals. In this study, 70% of patients with GD receiving velaglucerase alfa achieved all 6 therapeutic goals (hepatomegaly, splenomegaly, anemia, thrombocytopenia, bone pain, and bone crisis). The proportion of patients achieving each therapeutic goal was also high, ranging from 85 to 100%, and low plasma lyso-Gb1 concentration correlated with achievement of all 6 therapeutic goals. Furthermore, lyso-Gb1 concentration in this patient cohort was lower compared with that reported in a previous study of non-Japanese patients with type 1 GD receiving ERT. These results suggest that Japanese patients with GD are treated effectively with velaglucerase alfa, and that lyso-Gb1 concentration may be a useful biomarker to predict achievement of the treatment goals, which may ultimately be useful to also evaluate treatment efficacy for GD.

This study demonstrated that plasma lyso-Gb1 concentration in Japanese patients with GD treated with velaglucerase alfa is low, and although not statistically significant, low plasma lyso-Gb1 concentration correlates with a higher achievement rate of therapeutic goals for GD. Lower lyso-Gb1 concentration was observed in patients who had achieved all 6 therapeutic goals, assessed by the extent of hepatomegaly, splenomegaly, anemia (hemoglobin level), thrombocytopenia (platelet count), bone pain, and bone crisis, than in those who had not achieved all 6 therapeutic goals. Similarly, in a retrospective, exploratory analysis of data from phase 3 clinical trials of patients with type 1 GD treated with velaglucerase alfa, decreasing lyso-Gb1 concentrations had a statistically significant correlation with increasing platelet count and decreasing spleen volume in the treatment-naive group at some timepoints during treatment [21]. A previous cohort study of treated and untreated children with type 1 or 3 GD also reported that lyso-Gb1 significantly correlated with platelet count (*p* < 0.0001, r = − 0.42) and hemoglobin level (*p* = 0.003, r = − 0.35) [22]. Further, in a retrospective, multicenter observational study, 25 patients with GD who received an ERT (velaglucerase alfa [n = 17], imiglucerase [n = 4], or taliglucerase alfa [n = 4]) had decreased lyso-Gb1 levels and spleen and liver volumes, and increased platelet counts and hemoglobin levels [24]. Collectively, those results and the results of this study may further indicate that lyso-Gb1 is a useful biomarker to evaluate treatment response in patients with GD receiving ERT treatment. However, a target plasma lyso-Gb1 concentration to indicate treatment effectiveness has not yet been determined. Therefore, future studies to establish a target may be beneficial in guiding physicians when monitoring lyso-Gb1.

Lyso-Gb1 concentrations are expected to be high in Japanese patients with GD compared with non-Japanese patients with GD, as Japanese patients commonly have the L444P mutation [7, 8, 25], which is associated with a higher lyso-Gb1 level [19, 20], and a more severe phenotype of GD than other phenotypes [2, 19, 26, 27]. Baseline lyso-Gb1 concentrations were not available in this study, which is a limitation; however, in this study the median plasma lyso-Gb1 concentration observed in Japanese patients treated with velaglucerase alfa for a median duration of 49.5 months (~ 215.1 weeks) was 24.3 ng/mL, which was lower compared with those previously reported in non-Japanese patients [3, 21]. Results from a retrospective, exploratory analysis of patients with type 1 GD treated with velaglucerase alfa indicated that the median lyso-Gb1 concentration was 43.6 ng/mL at treatment week 209 for the treatment-naive patients, and 26.6 ng/mL at treatment week 161 for the switch-treatment patients [21]. In another study, the lyso-Gb1 concentration was 89 ng/mL, a reduction of 49% from 180.9 ng/mL, after a mean of 3.6 years (~ 187.7 weeks) of treatment with imiglucerase [3]. Possible reasons for the observation of lower lyso-Gb1 in this study of Japanese patients compared with the previous studies [3, 21] are that patients in this study were treated with velaglucerase alfa for a longer duration and possibly at a higher dose (60 U/kg every other week [approved dose in Japan] [28] versus 52.4 U/kg [treatment-naive patients] and 28.9 U/kg [switch-treatment patients] [21]). Furthermore, a previous study has reported a reduction in lyso-Gb1 concentration in 2 patients with type 3 GD treated with ambroxol chaperone therapy in combination with ERT [29]; given that the present study included 5 patients receiving ambroxol, the possibility of the effects of ambroxol in lowering lyso-Gb1 concentrations should not be excluded. Nonetheless, these findings demonstrate that lyso-Gb1 concentrations of ERT-treated patients are lower in Japanese patients with GD than non-Japanese patients with GD. Although patients’ baseline lyso-Gb1 concentrations were not available in this study, long-term treatment with velaglucerase alfa at a dose of 60 U/kg every other week may be effective in reducing lyso-Gb1 levels in Japanese patients with GD.

In this study, achievement rate of GD therapeutic goals was high (70%) in Japanese patients with GD who were treated with velaglucerase alfa, most likely at a dose of 60 U/kg every other week. A previous benchmark analysis of patients with type 1 GD treated with imiglucerase reported that the proportion of patients achieving all 6 GD therapeutic goals increased with higher doses of imiglucerase (achieved 1–3 goals: 37.6 U/kg/4 weeks; achieved 4 goals: 56.9 U/kg/4 weeks; 5 goals: 70.7 U/kg/4 weeks; all goals: 74.2 U/kg/4 weeks) [30]. Therefore, given that the dose of velaglucerase alfa was higher in this study (i.e., 120 U/kg/4 weeks), this may explain the high achievement rate of GD therapeutic goals observed in this study, further suggesting that higher doses of velaglucerase alfa treatment may be associated with a greater achievement rate of therapeutic goals.

In this study, although most patients had low plasma lyso-Gb1 concentrations, a plasma lyso-Gb1 concentration ≥ 100 ng/mL was observed in 2 patients. These results may be explained by the fact that 1 patient, who had type 1 GD, was treated with velaglucerase alfa for only 3 months, which is also reflected by the low treatment achievement rate (33.3%). The second patient had type 2 GD, was experiencing neurological and bone symptoms, and had developed immunoglobulin G and immunoglobulin E antibodies against both imiglucerase and velaglucerase alfa. The patient experienced serious infusion-associated reactions requiring treatment with antihistamines, corticosteroids, and analgesics prior to velaglucerase alfa administration. Nonetheless, results from this study suggest that velaglucerase alfa may be effective in achieving therapeutic goals for GD in Japanese patients, who generally have more severe disease compared with non-Japanese patients [9, 27].

In this study, the sample size was limited because the number of patients with GD is limited in Japan. The velaglucerase alfa dose used in this study and in previous studies varied; the approved dose of velaglucerase alfa in Japan is 60 U/kg every other week, which was higher than the dose used in previous studies conducted outside of Japan. Pre-treatment plasma lyso-Gb1 concentration was unavailable because the number of patients newly starting treatment was very small, and it is almost impossible to prospectively measure lyso-Gb1 concentration prior to treatment initiation in patients with GD in Japan. Therefore, the change in plasma lyso-Gb1 concentration from pre-treatment to post-treatment could not be evaluated. Furthermore, the study population was heterogeneous (included patients with type 1, 2, and 3 GD), making it difficult to distinguish whether higher levels of lyso-Gb1 reflect disease severity or low treatment response, and the study did not control for ambroxol use. Therefore, treatment-naive patients with GD should be enrolled in future studies to evaluate the correlation between the change in lyso-Gb1 concentration from pre-treatment phase and clinical parameters, including those 6 therapeutic goals selected in this study. Furthermore, patients treated with velaglucerase alfa for ≥ 3 months were included in this study based on our clinical experience that the effects of ERT stabilize at around 3–6 months; however, the results from this study indicated that ERT treatment with velaglucerase alfa for ≤ 3 months may be insufficient to stabilize lyso-Gb1 concentrations.

## Conclusions

In conclusion, this study demonstrated that low plasma lyso-Gb1 concentration correlates with higher achievement rate of therapeutic goals; lyso-Gb1 may be a useful biomarker for predicting the achievement of treatment goals, and for ultimately evaluating treatment response in patients with GD who are treated with ERT, including velaglucerase alfa. Further, the achievement rate of therapeutic goals in this study was high, and the plasma lyso-Gb1 concentration was lower compared with those previously reported in non-Japanese studies, indicating that velaglucerase alfa is an effective treatment for Japanese patients with GD.

## Methods

### Study design

This was a non-interventional, open-label, multicenter, observational cohort study to investigate the relationship between lyso-Gb1 concentration and therapeutic goals in Japanese patients with GD who were treated with velaglucerase alfa. The study was conducted at 14 sites between October 2020 and March 2021. The final data cut-off date was 19 March 2021. After enrollment, patients had one study site visit for data collection during the 6-month study period with regular administration of velaglucerase alfa; the enrollment and observational visit could have occurred in a single visit. The study was registered at the Japanese Registry of Clinical Trials (rctportal.niph.go.jp: jRCT1031200137). This study was conducted in accordance with the Declaration of Helsinki, Good Pharmacoepidemiology Practices, and local laws and regulations. All patients provided written informed consent before entering the study.

### Study population

Patients of any age were included in this study if they were diagnosed with GD type 1, 2, or 3 by a physician at any study site and had been treated with velaglucerase alfa for ≥ 3 months. Patients who were, in the study investigator’s opinion, unsuitable to participate were excluded from this study.

### Outcome measures

The primary outcome was plasma lyso-Gb1 concentrations by overall achievement rate of 6 therapeutic goals (improvements in hepatomegaly, splenomegaly, anemia, thrombocytopenia, bone pain, and bone crisis). Secondary outcomes were plasma lyso-Gb1 concentration by GD type, and by pathological mutation type. The exploratory endpoint was the comparison of plasma lyso-Gb1 concentrations in Japanese patients with GD compared with lyso-Gb1 concentrations reported in a previous study of non-Japanese patients with GD receiving an ERT [21].

### Data collection and assessments

Patient demographics and baseline characteristics, including age, sex, duration of disease, disease type, duration of velaglucerase alfa treatment, and *GBA* gene mutation type, were collected at enrollment. Genotyping was performed at each study site; a patient’s genetic information was reviewed and confirmed by the pediatrician. Plasma lyso-Gb1 concentration was measured in blood samples collected as part of routine medical care, using a liquid chromatography with tandem mass spectrometry (LC–MS/MS) method at LSI Medience Corporation (Tokyo, Japan). Briefly, 3 mL of blood was collected in EDTA-2K, which was centrifuged at 2000–3000 g for 15 min at 4 °C; 1 mL of supernatant was then stored at − 80 °C. Six therapeutic goals (improvements in hepatomegaly, splenomegaly, anemia, thrombocytopenia, bone pain, and bone crisis) were measured as part of routine standard of care at each study site, at enrollment or at the observational visit. The degree of achievement of each therapeutic goal was assessed using modified scales from previous reports [30, 31]. Hepatomegaly and splenomegaly were assessed using a scale of 0–3; 0 indicating achievement of therapeutic goal; 1 indicating the liver/spleen is palpable, but its margin does not cross the navel; 2 indicating the limb of liver/spleen is palpated between the umbilicus and the iliac crest, but for spleen, it does not reach the right abdomen; and 3 indicating the liver/spleen reaches beyond the iliac crest to the pelvic cavity and practically occupies the entire abdomen. For anemia, the therapeutic goal was achieved if the hemoglobin level was ≥ 11.0 g/dL for children aged ≤ 12 years and females ≥ 13 years, and ≥ 12.0 g/dL for males ≥ 13 years of age. For thrombocytopenia, the therapeutic goal was achieved if platelet count was > 120 × 10^3^/μL for those with platelet count ≥ 60 × 10^3^/μL at the first infusion of velaglucerase alfa, or with no data available at the first velaglucerase alfa infusion, or if ≥ 2 times the platelet count of the first velaglucerase alfa infusion for those with platelet count < 60 × 10^3^/μL at the first infusion of velaglucerase alfa. Bone pain was assessed as “absent or very mild pain,” “mild pain,” “moderate pain,” “severe pain,” and “unbearable pain”; the therapeutic goal was achieved if “absent or very mild pain” or “mild pain” was reached. Furthermore, the presence and absence of bone crisis was assessed, with its absence indicating achievement of therapeutic goal. Adverse events were reported as part of routine clinical practice at enrollment and/or observational visit, and at discontinuation; the causal relationship was determined by the treating physician at each study site.

### Statistical analysis

No target sample size was set for this study because in Japan there are a limited number of patients diagnosed with GD [6]. All assessments were conducted using the full analysis set, defined as all enrolled patients who were examined at least once for either of the study endpoints. Patient demographics and baseline characteristics were summarized using descriptive statistics. Categorical variables are presented as n (%), and continuous variables are presented as mean (standard deviation [SD]), and/or median (minimum [min]–maximum [max]; interquartile range). Lyso-Gb1 concentrations were summarized for all patients, by overall achievement rate of therapeutic goals (100% achievement [all 6 therapeutic goals achieved] versus not 100% achievement [all 6 therapeutic goals not achieved]), by disease type, and by mutation type, using descriptive statistics. Between-group comparison was conducted using Wilcoxon rank sum test; *p* < 0.05 was considered statistically significant. No safety analysis was performed. Statistical analyses were performed using SAS version 9.4 (Cary, NC, USA).

## Supplementary Information

**Additional file 1: Fig. S1**. Achievement rate of therapeutic goals.

## Data Availability

The datasets used and/or analyzed during the current study are available from the corresponding author on reasonable request.

## Abbreviations

ERT: Enzyme replacement therapy
GD: Gaucher disease
*GBA*: β-Glucocerebrosidase gene
LC–MS/MS: Liquid chromatography with tandem mass spectrometry
lyso-Gb1: Glucosylsphingosine
