# Supplementary material for: An observational study to investigate the relationship between plasma glucosylsphingosine (lyso-Gb1) concentration and treatment outcomes of patients with Gaucher disease in Japan
Source: Orphanet J Rare Dis. 2022 Nov 3;17:401. doi: 10.1186/s13023-022-02549-6 (PMC9635088; doi:10.1186/s13023-022-02549-6)

Additional file 1

**Additional file 1: Fig. S1.** Achievement rate of therapeutic goals.

Note: Therapeutic goals for hepatomegaly and splenomegaly assessments were achieved if patients had a score of 0 (no palpable liver or spleen). Therapeutic goals for anemia and thrombocytopenia assessments were achieved if hemoglobin level was ≥11.0 g/dL for children ≤12 years and females ≥13 years, and ≥12.0 g/dL for males ≥13 years, and if platelet count was >120 x 10^3^/μL for those with platelet count ≥60 x 10^3^/μL at the first infusion of velaglucerase alfa, or with no data available at the first velaglucerase alfa infusion, or ≥2 times the platelet count at first velaglucerase alfa infusion for those with platelet count <60 x 10^3^/μL at the first infusion of velaglucerase alfa. Therapeutic goals were achieved for bone pain and bone crisis assessments if patient had no to mild pain, and no bone crisis. N=20.


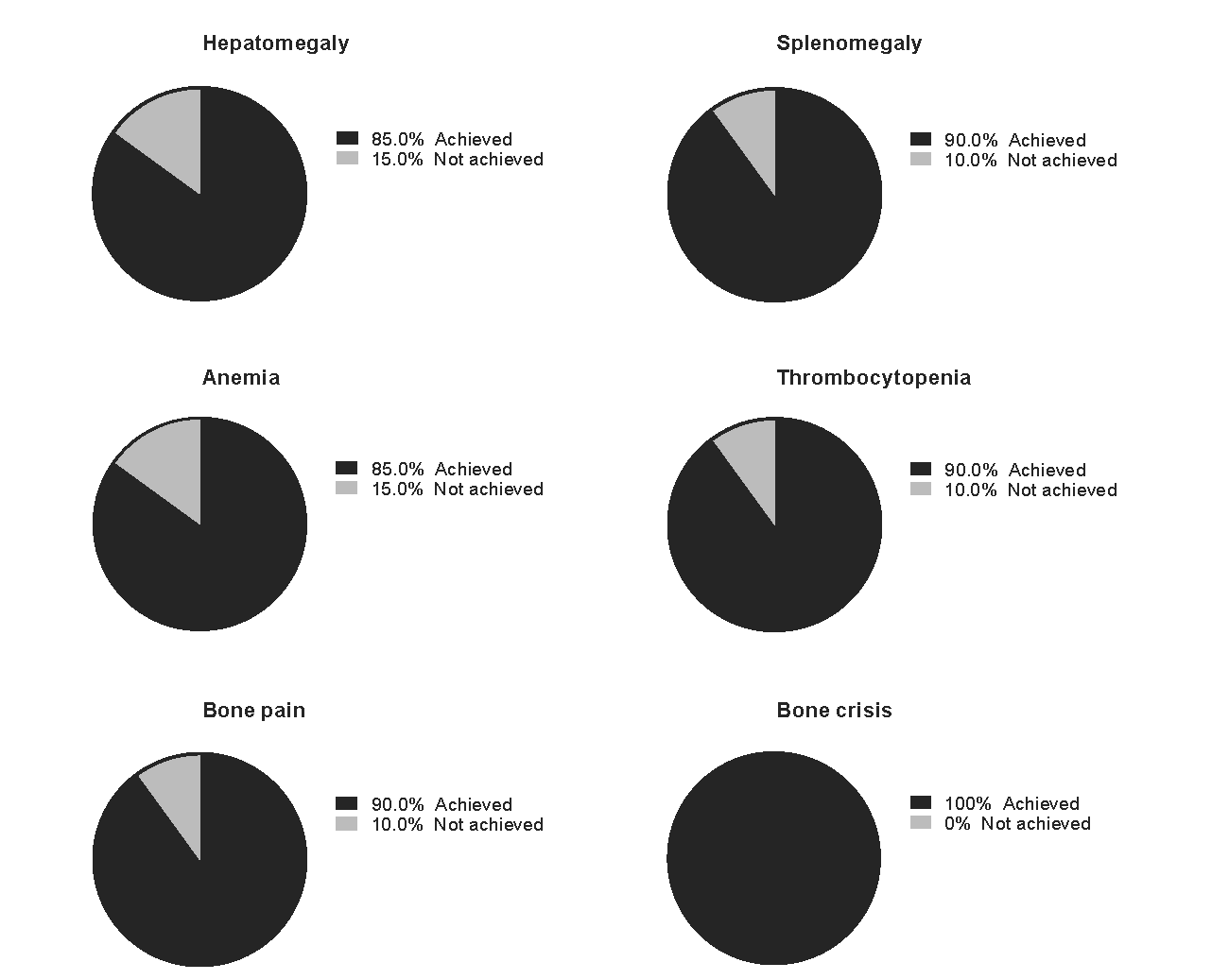

Supplement: Supplementary file 1 — Additional file 1: Fig. S1. Achievement rate of therapeutic goals. [file 13023_2022_2549_MOESM1_ESM.docx]
